# Supplementary material for: Solutions for decarbonising urban bus transport: a life cycle case study in Saudi Arabia
Source: Commun Eng. 2024 Jul 9;3:95. doi: 10.1038/s44172-024-00238-9 (PMC11233610; doi:10.1038/s44172-024-00238-9)
Supplement: Supplementary file 2 — Supplementary Information [file 44172_2024_238_MOESM2_ESM.pdf]

Supplementary Information for Solutions for Decarbonizing Urban Bus Transport in non-OECD Countries: A Life Cycle Case Study in Saudi Arabia

Chengcheng Zhao[1], Leiliang Zheng Kobayashi[1], Awad Bin Saud Alquaity[2,3], Jean-Christophe Monfort[4], Emre Cenker[4], Noliner Miralles[5] and S. Mani Sarathy[1*]

[1]Clean Combustion Research Center (CCRC), King Abdullah University of Science and Technology (KAUST), Thuwal, 23955, Saudi Arabia.
[2]Department of Mechanical Engineering, King Fahd University of Petroleum & Minerals (KFUPM), Dhahran, 31261, Saudi Arabia.
[3]Center for Hydrogen and Energy Storage, King Fahd University of Petroleum & Minerals (KFUPM), Dhahran, 31261, Saudi Arabia.
[4]Transport Technologies Division, R&DC, Saudi Aramco, Dhahran, 31311, Saudi Arabia.
[5]Saudi Public Transport Company (SAPTCO), Riyadh, 11443, Saudi Arabia.

*Corresponding author(s). E-mail(s): mani.sarathy@kaust.edu.sa; Contributing authors: chengcheng.zhao@kaust.edu.sa; leiliang.kobayashi@kaust.edu.sa; awad.alquaity@kfupm.edu.sa; jeanchristophe.monfort.1@aramco.com; emre.cenker@aramco.com; mirallesnc@saptco.com.sa;

Supplementary Notes

I Supplementary Note 1: Vehicle cycle

I.1 The bill of buses materials-buses material inventory

Specifications information for three types of buses, combining information from the literature, company consultation, and other publicly available data[1–5], as shown in Supplementary Table 1.

Supplementary Table 1 Specifications of buses[1,6–8].

	ICEB	BEB	PEM FCB
Weight (kg)	12,700	14,400	12,464
Dimensions			
Length (m)	12.5	12.5	10.5
Width (m)	2.5	2.6	2.5
Height (m)	2.3	3.4	3.3
Capacity	35 seated	37 seated	22 seated
Motor			
Engine/motor type<br>Max. power (kW)	8L inline 6-cylinder diesel engine	AC Synchronous	AC synchronous
Battery			
Battery type	Lead	LFP	NiMH
Battery capacity<br>power supply amount	165 Ah	313 kWh	235 kWh
Fuel cell stack and hydrogen tank			
Fuel cell stack, max output	-	-	114 kW x 2
hydrogen tank, pressure	-	-	70 MPa x 10
High-pressure hydrogen tank volume (l)			600
Refuelling times	10min	2-2.5 hrs (150kW)	6-8min
Front tyre	275/70 R22.5	275/70 R22.5	275/70 R22.5
Rear tyre	295/80 R22.5	455/45 R22.5	455/45 R22.5

I.2 Buses transportation - shipping

Supplementary Table 2 presents the emission factors for shipping.

Supplementary Table 2 Emission factors for main engines, auxiliary engines, and auxiliary boilers in heavy fuel oil-fuelled vessels.[9,10]

Emissions	kg per t
NO_x	71.80
SO_2	58.18
CO_2	3433.30
PM	8.03
NMVOC	3.40
CH_4	0.07
N_2O	0.18
CO	3.05
BC	0.18
OC	0.61

I.3 Replacement information

Supplementary Table 3 elucidates the replacement details for each bus component.

Supplementary Table 3 Vehicle component replacement frequencies correlated to kilo-metric usage.

components	lifetime	references
Powertrain Coolant	62,500 km	[11]
Transmission Fluid	60,000 km	[12]
Brake Fluid	62,500 km	[11]
Windshield Fluid	12,500 km	[11]
Tire	80,000 km	[12]
Engine Oil	6,250 km	[11]
LFP Battery	6,000 cycles	[13]
NiMH battery	3,000 cycles	[13]
Lead-acid battery	2,000 cycles	[1,11,12]
PEM FC stack	50,000-80,000 h	[14]

II Supplementary Note 2: Impact of air conditioning (AC) systems on bus operating phase and calculation

The phase in which the fuel cycle and vehicle cycle intersect is referred to as the bus operation stage, commonly known as the tank-to-wheel stage. Owing to Saudi Arabia's unique geographical location, outdoor temperatures reach exceptional highs, particularly in Makkah, where the buses under investigation in this study operate at an average annual temperature of 39 °C. One research objective is to assess the influence of energy consumption during bus operation at such elevated temperatures on the overall life-cycle assessment. Consequently, this study refined the methodology for calculating emissions during the operational phase, enabling a comprehensive and detailed approach to determine the cooling load of bus AC systems.

Supplementary Table 4 Characterisation of bus elements: detailed information on areas, K, and SC for various components. [15]

Surface	Area (m^2)	K-Value Wm^{-2}K^{-1}	Shade coefficient (SC)
Body			
Roof	32	1.87	
Floor	33	2.267	
Side panel	24	2.801	
Front body	2	2.667	
Rear body	2	2.667	
Glass			
Front window	3	5.02	0.811
Rear window	3	2.611	0.811
Side window	26	2.569	0.811
Driver windows	2	4.89	0.811
Other			
Door	2	4.89	0.811
Skylight	1	4.89	0.811

Supplementary Table 5 The pump-to-wheel energy conversion efficiency for FCBs, BEBs, and ICEBs Technologies. [16–21]

	ICEB	BEB	PEM FCB
η_{engine}	0.35		
$\eta_{mechanical\_transmission}$	0.85		
η_{charge}		0.9	
$\eta_{battery}$		0.92	
$\eta_{inverter}$		0.96	0.96
η_{motor}		0.93	0.93
η_{fuel_cell}			0.518
$\eta_{dispenser/charger}$		0.985	0.92

Supplementary Table 6 Energy consumption from tank-to-wheel (kWhkm^{-1}).

	ICEB	BEB	PEM FCB
Total energy consumption	5.16	1.60	3.25

III Supplementary Note 3: Hydrogen cycle

Generated in SMR plants, hydrogen typically functions at low-pressure levels of 20-30 bar, necessitating compression to 200-500 bar for transportation purposes[22–24]. Hydrogen is conveyed from the SMR plant to the bulk terminal via a pipeline, exhibiting an energy intensity of 4590 btu ton^{-1} mile^{-1} and spanning a 1.6 km distance. A centrifugal compressor, characterised by an 80% efficiency, subsequently raises the hydrogen pressure from 30 bar to 350 bar, facilitating transport using a gas tube trailer with a 450 kg capacity and a 350 bar pressure[23–25]. According to the GREET database, energy intensities of 17,920 btu ton^{-1} mile^{-1} and 14,222 btu ton^{-1} mile^{-1} correspond to trailer transport between the SMR plant and RFS, and in the reverse direction, respectively. Two unique types of hydrogen—blue hydrogen procured from Jubail, located 1333 km away, and grey hydrogen from Yanbu, 360 km distant—are transported from their individual SMR plants to the Makkah RFS.

To ensure safe hydrogen compression and storage at the Makkah RFS, the GREET model ascertained a necessary compression level of 925 bar, integrating a 1.25 safety factor and a 50-bar pressure differential between the pipeline trailer and the RFS. Employing a reciprocating compressor with a 65% efficiency, the hydrogen undergoes compression before being stored in high-pressure storage units situated at the RFS[26]. Supplementary Table 8 delineates the material breakdown for the hydrogen RFS. Moreover, pre-cooling was implemented to lower the hydrogen temperature to -40°C, mitigating the risk of overheating. The cooling load of the pre-cooling system was determined based on Swidler's 2020 model[24]. Given these parameters, the G.H_2 compression efficiency at RFSs was found to be 92.8%. The hydrogen flow rate channelled into the bus-quick fill dispenser complied with the SAE J2601/2 standard's safety constraints, exhibiting a maximum flow rate of 0.12 kg s^{-1}[27].

III.1 Loss factors associated with hydrogen transport.

Supplementary Table 7 shows the loss factors associated with hydrogen transport.

Supplementary Table 7 Loss factor of hydrogen transportation.

Process	Factor	Source
Liquefaction	1.005	HDSAM[28]
G.H2 pipeline	1.005	HDSAM[28]
G.H2 bulk terminal	1.01	HDSAM[28]
Gas tube trailer	1.05	[23]
RFS for G. H2	1.005	HDSAM[28]

III.2 CCS methods used in blue hydrogen production and efficiency

The SMR process is a well-established technology for hydrogen production. This study investigates the energy consumption and pollutant emissions associated with blue and grey hydrogen production using SMR. To reduce the carbon footprint of the production process, blue hydrogen production employs CCS technology to capture CO_2 emissions from combustion and reforming in NG SMR plants.

Equation (S1) defines the CO_2 capture efficiency[29].

For the 2030 scenario, a CCS efficiency of 96.2% was assumed, which was achieved by combining pre-combustion CO_2 separation technologies such as water gas transfer reactors with solvent-based CO_2 separation technologies like methyl diethanolamine (MDEA) and Shell Cansolv systems to achieve high CO_2 separation rates[29].

$$\text{Carbon capture efficiency} = (1-(\frac{\text{Carbon in stack and CO}_2\text{ dryer vent}}{\text{Total carbon in}}))*100\% \tag{S1}$$

III.3 Hydrogen refuelling station infrastructure

The designed hydrogen refuelling station (RFS) has a capacity of 94,500 kg of hydrogen per year, including necessary infrastructure components such as building foundations and compressors, as outlined in Supplementary Table 8[30].

Supplementary Table 9 provides crucial data on the expected service life of the essential components of the RFS infrastructure, including RFS, compressors, storage tanks, trailers, and hydrogen pipelines. The study assumed a 10-year lifetime for the entire bus fleet. All the components mentioned above are expected to have a service life of at least 10 years. Therefore, it can be inferred that the missing components of the fuelling station infrastructure need not be replaced within the specified 10-year period.

Supplementary Table 8 The material breakdown list of hydrogen RFS established in Makkah with the annual hydrogen capacity of 94,500 kg.

Building of foundations	kg
Steel	557.36
Smooth coated glass	201.00
Plaster fibre plate	6.18
Silica sand	3,563.54
Concrete	0.62
Resistant concrete	8.04
Gravel	111,470.44
Lubricant oil	1.24
Storage module	kg
Stainless Steel 18/8	5,204.88
Other components	kg
Steel	101.82
Nitrogen	8.86
Stainless Steel 18/8	25.02
Polypropylene, granulated	0.62
Compressor	kg
Steel	153.60
Stainless Steel 18/8	117.62
Casting iron	37.12
Etilenoglicol	0.44
Lubricant oil	1.12
Aluminium	3.72
Insulation tube	0.94
Copper	2.78

Supplementary Table 9 The lifetime of components in the hydrogen RFS.[22,30–32]

Components	Duration of life in year
Fuel station	15
Dispenser	10
Compressor	10
Storage	30
Trailer	30
Pipeline	50

IV Supplementary Note 4: Electricity cycle

IV.1 Gogeospatial visualisation

Supplementary Figure 1 shows the geospatial visualisation and analysis of oil and gas fields, power plants, and bulk plants in Saudi Arabia using multi-source data.

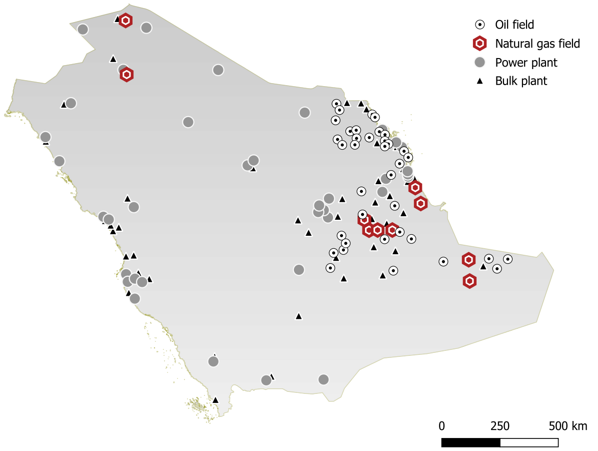

Supplementary Figure 1 Geospatial Visualisation and Analysis of Oil and Gas Fields, Power Plants, and Bulk Plants in Saudi Arabia using Multi-Source Data, 2021 [33]. Geospatial Information on Subnational Administrative Boundaries in Saudi Arabia was obtained from open sources [34].

IV.2 Power Plant Composition, Efficiency, and Emission Factors in Saudi Arabia

The distribution of technological configurations in oil and gas power generation is shown in Supplementary Figure 2. It illustrates the distribution of different types of plant.

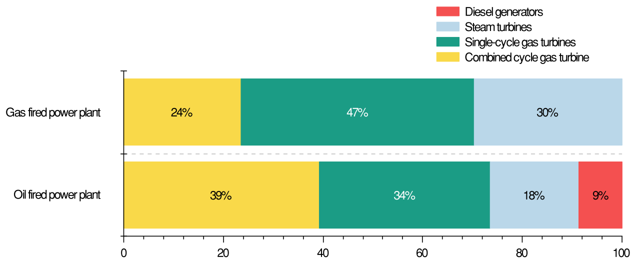

Supplementary Figure 2 Distribution of technological configurations in oil and NG power generation, showcasing the proportions of conventional NG turbines, combined cycle gas turbines, steam turbines, single cycle gas turbines, and diesel generators.

Supplementary Table 10 show the CO_2 emission factors for various power plant technologies.

Supplementary Table 10 CO_2 emission factors for various power plant technologies, encompassing conventional NG turbines, combined cycle gas turbines, steam turbines, single cycle gas turbines, and diesel generators.

Fuel	Combustion technology	CO_2 emission factor (kg_{CO_2} kWh^{-1})[1]
Crude	gas turbine	0.76
	combined-cycle	0.48
	steam-turbine	0.67
	diesel generator	0.80[2]
Diesel	gas turbine	0.76
	diesel generator	0.76
Gas	conventional gas turbine	0.68[3]
	combined-cycle	0.37[3]
HFO	steam-turbine	1.00[3]
	diesel generator	1.00[3]

[1]The CO_2 emissions factors in this study are primarily derived from the CO_2 footprint database[35], with the exception of crude oil diesel generators, heavy fuel oil-fired power plants, and NG-fired power plants, which utilise data from the ecoinvent database.

[2]Literature-based information on crude oil diesel generators is sourced from[36].

[3]Data utilised from the ecoinvent database are specific to the context of Saudi Arabia.

The share of the Saudi grid mix is shown in Fig. Supplementary Figure 3.

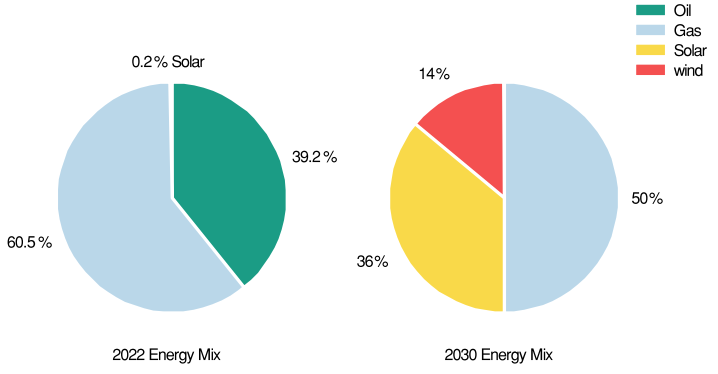

Supplementary Figure 3 Transition in Saudi Arabia's electricity generation energy mix between 2022 and 2030, highlighting the dominance of fossil fuels in 2022 and the targeted increase in renewable energy share to 50% by 2030.

IV.3 Quantification of emissions and energy consumption from super-fast charging stations (SFCS)

The material composition breakdown is presented in Supplementary Table 11, with the material production emissions factor derived from the GREET database.

Supplementary Table 11 Material breakdown of an electric vehicle charging station[20].

Material	weight in kg
Aluminium	554.6
Copper	2023.2
Glass	1.2
Plastic	190
Steel	630
Ethylene, Glycol	658.2
Rubber	22.8

122 # V Supplementary Note 5: Diesel cycle

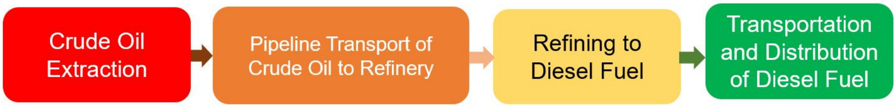

Supplementary Figure 4 Flow Chart of Diesel Production and Transportation to Retail Fuel Stations.

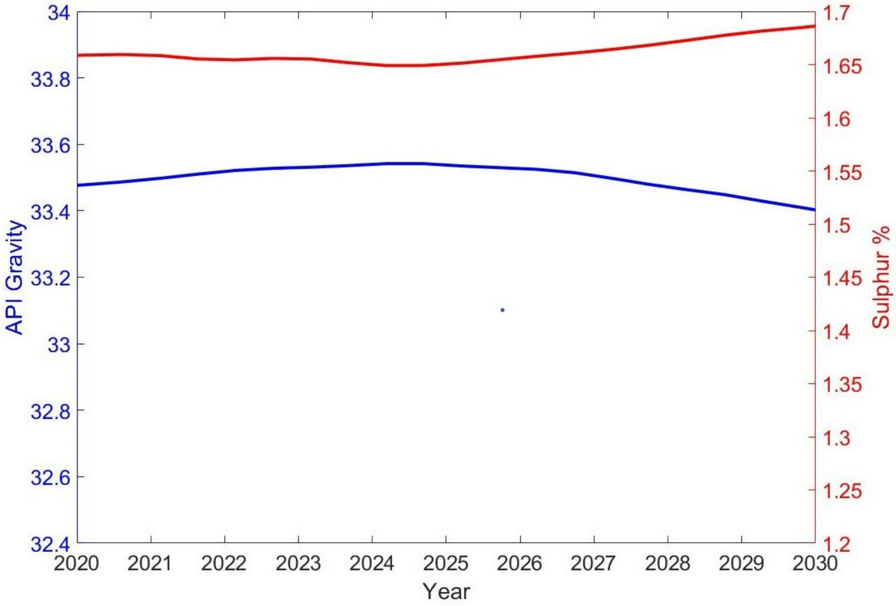

Supplementary Figure 5 Average OPEC Crude Oil Quality from 2020 to 2030.

VI Supplementary Note 6: 2030 scenario-bus weight

The estimated weights of the blue and grey FCBs, BEBs, and ICEBs in 2030 are listed in Supplementary Table 12.

Supplementary Table 12 The bus weight in 2022 and 2030 scenario.

Year	ICEB	BEB	FCB
2022	12,700	14,400	12,464
2030	9,457	11,483	9,939

VII Supplementary Note 7: life-cycle impact assessment

In this study, the CML2001-LCIA method was implemented for life-cycle impact assessment (LCIA). The characterisation factor for the LCIA, as delineated in Supplementary Table 13. The results of the 2030 scenario are shown in Supplementary Figure 6.

Supplementary Table 13 LCIA characterisation factor.

	GWP100	GWP20	EP	AP	POP
CO	-	-	-	-	0.027
CO_2	1	1	-	-	-
CH_4	28	84	-	-	0.006
N_2O	265	264	0.27	-	-
NO_x	-	-	0.13	0.5	0.028
SO_2	-	-	-	1.2	0.048

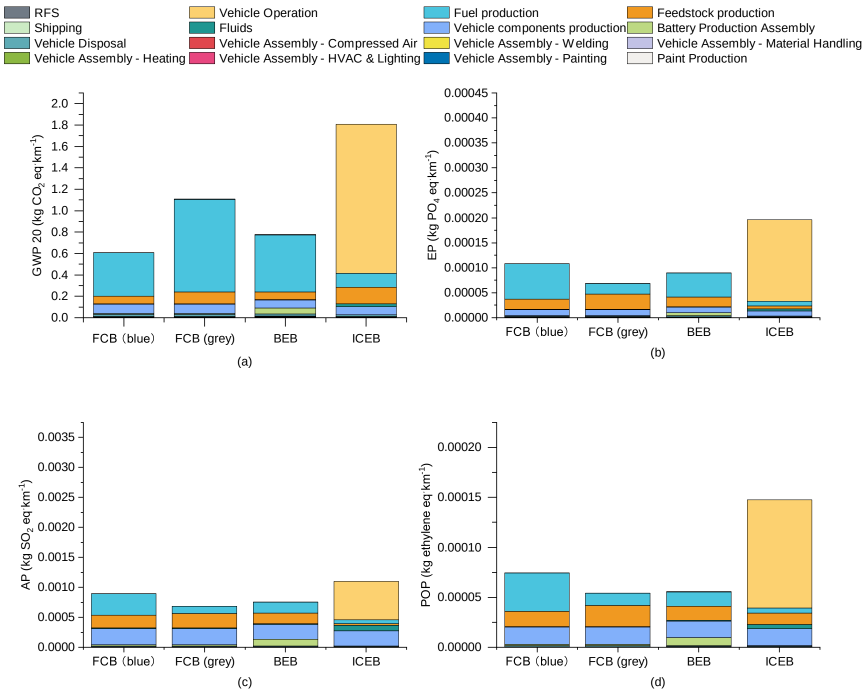

Supplementary Figure 6 Life cycle impact analysis (LCIA) results showcasing the 20-year global warming potential (GWP20), eutrophication potential (EP), acidification potential (AP), and photochemical oxidation potential (POP) calculated using the CML2001 methodology. This comprehensive analysis spans the entire life cycle of buses in 2022, comparing blue and grey hydrogen fuel cell buses (FCB), battery electric buses (BEB), and internal combustion engine buses (ICEB). Panels (a) to (d) illustrate the impacts on GWP20, EP, AP, and POP, respectively. RFS stands for refuelling station; HVAC stands for heating, ventilation, and air conditioning.

VIII Supplementary Note 8: Energy consumption results presented in basic unit

Supplementary Table 14 Comparative lifecycle energy consumption of various bus models in 2022 and 2030 Scenarios (Measured in MJ).

	Blue FCB	Blue FCB	Grey FCB	Grey FCB	BEB	BEB	ICEB	ICEB
Scenario	2022	2030	2022	2030	2022	2030	2022	2030
ADR	516,231.2	228,267.2	516,231.2	228,267.2	596,416.0	263,723.3	526,005.8	217,209.4
Battery production assembly	72,019.2	66,496.5	72,019.2	66,496.5	368,265.9	329,573.1	1,812.8	1,596.3
Vehicle components production	774,398.7	623,078.7	774,398.7	623,078.7	680,879.0	517,661.7	668,338.9	511,525.2
Fluids production	80,305.6	77,187.0	80,305.6	77,187.0	92,779.5	89,176.5	253,509.7	249,921.6
Shipping	31,867.6	0.0	31,867.6	0.0	29,780.2	0.0	24,280.5	0.0
Feedstock production	397,349.3	370,287.4	447,665.4	416,140.4	679,688.4	303,618.4	186,196.2	159,421.4
Fuel production	6,709,114.3	5,721,988.4	2,886,467.6	2,160,714.5	5,971,490.1	2,898,131.5	1,059,962.9	1,022,696.7
Vehicle operation	5,940,898.5	5,550,955.4	5,940,898.5	5,550,955.4	2,978,757.8	2,978,757.8	9,437,838.8	9,437,838.8
RFS	1,706.8	1,292.4	1,706.8	1,292.4	18,270.2	15,640.6	0.0	0.0
Total	14,523,891.3	12,639,553.0	10,751,560.7	9,124,132.0	11,416,327.1	7,396,282.9	12,157,945.6	11,600,209.4

IX Supplementary Note 9: Energy intensity of fuel transportation

The energy intensity of fuel transportation is presented in Supplementary Table 15.

Supplementary Table 15 Energy intensity of fuel transportation.

Energy Intensity of Transportation	Btu ton^{-1} $mile^{-1}$	References
Crude Pipeline	260	[37]
Residual Oil Pipeline	260	[37]
Diesel Pipeline	260	[37]
NG Pipeline	567	[17]
Gaseous Hydrogen Pipeline	4,590	GREET
Tube trailer (origin to RFS)	17,920	GREET
Tube trailer (back-haul)	14,222	GREET

X Supplementary Note 10: Life cycle energy consumption results and GHG emissions per functional unit.

Supplementary Table 16 The energy consumption throughout the life-cycle in the scenarios projected for 2022 and 2030, quantified in megajoules (MJ) per kilometer. RE*: renewable energy.

	Blue FCB	Blue FCB	Grey FCB	Grey FCB	BEB	BEB	ICEB	ICEB	BEB RE	Mixed FCB	BEB 100%RE*
Life cycle stages	2022	2030	2022	2030	2022	2030	2022	2030	2022	2030	2030
ADR	1.016	0.449	1.016	0.449	1.174	0.519	1.035	0.428	1.174	0.449	0.519
Battery Production Assembly	0.142	0.131	0.142	0.131	0.725	0.649	0.004	0.003	0.725	0.131	0.649
Vehicle components production	1.524	1.226	1.524	1.226	1.340	1.019	1.315	1.007	1.340	1.226	1.019
Fluids production	0.158	0.152	0.158	0.152	0.183	0.176	0.499	0.492	0.183	0.152	0.176
Shipping	0.063	0.000	0.063	0.000	0.059	0.000	0.048	0.000	0.059	0.000	0.000
Total vehicle cycle	2.903	1.958	2.903	1.958	3.480	2.362	2.901	1.929	3.480	1.958	2.362
Feedstock production	0.782	0.729	0.881	0.819	1.338	0.598	0.366	0.314	0.000	0.731	0.000
Fuel production	13.205	11.262	5.681	4.253	11.753	5.704	2.086	2.013	0.441	11.122	0.441
Vehicle Operation	11.693	10.925	11.693	10.925	5.863	5.863	18.575	18.575	5.863	10.925	5.863
RFS	0.003	0.003	0.003	0.003	0.036	0.031	0.000	0.000	0.036	0.003	0.031
Total fuel cycle	25.683	22.919	18.258	16.000	18.990	12.195	21.028	20.902	6.340	22.780	6.335
Total life-cycle	28.586	24.877	21.161	17.958	22.470	14.557	23.929	22.831	9.820	24.739	8.697

Supplementary Table 17 The life-cycle GHG emissions for the 2022 and 2030 scenarios, expressed in $kgCO_2$eq. per km. RE*: renewable energy.

	Blue FCB	Blue FCB	Grey FCB	Grey FCB	BEB	BEB	ICEB	ICEB	BEB RE	Mixed FCB	BEB 100%RE*
Life cycle stages	2022	2030	2022	2030	2022	2030	2022	2030	2022	2030	2030
ADR	0.087	0.030	0.087	0.030	0.100	0.034	0.089	0.028	0.100	0.030	0.034
Battery Production Assembly	0.008	0.007	0.008	0.007	0.052	0.045	0.000	0.000	0.052	0.007	0.045
Vehicle components production	0.107	0.081	0.107	0.081	0.098	0.071	0.094	0.068	0.098	0.081	0.071
Fluids production	0.005	0.004	0.005	0.004	0.005	0.005	0.027	0.026	0.005	0.004	0.005
Shipping	0.000	0.000	0.000	0.000	0.000	0.000	0.000	0.000	0.000	0.000	0.000
Total vehicle cycle	0.206	0.122	0.206	0.122	0.256	0.155	0.209	0.122	0.256	0.122	0.155
Feedstock production	0.058	0.054	0.077	0.071	0.121	0.048	0.077	0.072	0.000	0.054	0.000
Fuel production	0.577	0.385	1.011	0.851	1.128	0.533	0.133	0.127	0.000	0.394	0.000
Vehicle Operation	0.000	0.000	0.000	0.000	0.000	0.000	1.393	1.393	0.000	0.000	0.000
RFS	0.000	0.000	0.000	0.000	0.002	0.002	0.000	0.000	0.002	0.000	0.002
Total fuel cycle	0.636	0.439	1.089	0.922	1.251	0.583	1.603	1.592	0.002	0.449	0.002
Total life-cycle	0.842	0.561	1.295	1.044	1.507	0.738	1.812	1.714	0.258	0.571	0.157

Supplementary References

[1] Zhao, E., Walker, P.D., Surawski, N.C.: Emissions life cycle assessment of diesel, hybrid and electric buses. Proc. Inst. Mech. Eng. Pt. D J. Automobile Eng. **236**(6), 1233–1245 (2022)

[2] Company, C.G.C.: The Prius Battery Pack. Clean greencar. Accessed: 19 June 2023 (2008). http://www.cleangreencar.co.nz/page/prius-battery-pack

[3] InsideEVs: Toyota Mirai Fuel Cell Sedan Priced At $57,500: Specs, Videos. Insideevs. Accessed: 19 June 2023 (2015). https://insideevs.com/news/323973/toyota-mirai-fuel-cell-sedan-priced-at-57500-specs-videos/

[4] Toyota: The New Toyota Mirai. Toyota Europe Newsroom. Accessed: 19 June 2023 (2020). https://newsroom.toyota.eu/the-new-toyota-mirai/

[5] Wang, M., Elgowainy, A., Lu, Z., Baek, K.H., et al.: Greenhouse gases, Regulated Emissions, and Energy use in Technologies Model ® (2022 .Net). [Computer Software] https://doi.org/10.11578/GREET-Net-2022/dc.20220908.2 (2022)

[6] BYD Auto: K9M - 40' TRANSIT BUS. BYD. Accessed on 19 April 2023 (2019). https://en.byd.com/bus/k9m/

[7] Volvo Buses Global: Specifications Volvo B8R Low Entry. Volvo. Accessed on 19 April 2023 (2020). https://www.volvobuses.com/en/city-and-intercity/chassis/volvo-b8r-le/specifications.html

[8] Toyota: Toyota Launches Production Model "Sora" FC Bus. Toyota. Accessed on 19 April 2023 (2018). https://global.toyota/en/newsroom/corporate/21863761.html

[9] Bond, T.C., Bhardwaj, E., Dong, R., Jogani, R., Jung, S., Roden, C., Streets, D.G., Trautmann, N.M.: Historical emissions of black and organic carbon aerosol from energy-related combustion, 1850-2000. Atmospheric Chemistry and Physics Discussions **8**, 18323–18373 (2008). https://doi.org/10.5194/acpd-8-18323-2008

[10] International Maritime Organization: Third IMO Greenhouse Gas Study 2014: Executive Summary and Final Report. IMO (2014). https://greenvoyage2050.imo.org/wp-content/uploads/2021/01/third-imo-ghg-study-2014-executive-summary-and-final-report.pdf

[11] Chen, Y., Hu, X., Liu, J.: Life cycle assessment of fuel cell vehicles considering the detailed vehicle components: comparison and scenario analysis

in china based on different hydrogen production schemes. Energies **12**(15), 3031 (2019)

[12] Yang, L., Yu, B., Yang, B., Chen, H., Malima, G., Wei, Y.-M.: Life cycle environmental assessment of electric and internal combustion engine vehicles in china. J. Clean. Prod. **285**, 124899 (2021)

[13] Majeau-Bettez, G., Hawkins, T.R., Strømman, A.H.: Life cycle environmental assessment of lithium-ion and nickel metal hydride batteries for plug-in hybrid and battery electric vehicles. Environ. Sci. Technol. **45**(10), 4548–4554 (2011)

[14] Aydin, M.I., Dincer, I., Ha, H.: Development of oshawa hydrogen hub in canada: A case study. Int. J. Hydrogen Energy **46**(47), 23997–24010 (2021)

[15] Tosun, E., Bilgili, M., Tuccar, G., Yasar, A., Aydin, K.: Exergy analysis of an inter-city bus air-conditioning system. International Journal of Exergy **20**(4), 445–464 (2016)

[16] Basma, H., Mansour, C., Haddad, M., Nemer, M., Stabat, P.: Comprehensive energy assessment of battery electric buses and diesel buses. In: 32nd International Conference on Efficiency, Cost, Optimization, Simulation and Environmental Impact of Energy Systems (2019)

[17] Li, M., Zhang, X., Li, G.: A comparative assessment of battery and fuel cell electric vehicles using a well-to-wheel analysis. Energy **94**, 693–704 (2016)

[18] Pollet, B.G., Staffell, I., Shang, J.L.: Current status of hybrid, battery and fuel cell electric vehicles: From electrochemistry to market prospects. Electrochimica Acta **84**, 235–249 (2012)

[19] Suh, I.-S., Lee, M., Kim, J., Oh, S.T., Won, J.-P.: Design and experimental analysis of an efficient hvac (heating, ventilation, air-conditioning) system on an electric bus with dynamic on-road wireless charging. Energy **81**, 262–273 (2015)

[20] Zhao, E., May, E., Walker, P.D., Surawski, N.C.: Emissions life cycle assessment of charging infrastructures for electric buses. Sustain. Energy Technol. Assess. **48**, 101605 (2021)

[21] Smith, D., Ozpineci, B., Graves, R.L., Jones, P., Lustbader, J., Kelly, K., Walkowicz, K., Birky, A., Payne, G., Sigler, C., et al.: Medium-and heavy-duty vehicle electrification: An assessment of technology and knowledge gaps. Technical report (2020). https://info.ornl.gov/sites/publications/Files/Pub136575.pdf

[22] Hancke, R., Danebergs, J., Kvalbein, L., Aarskog, F.: Efficient hydrogen infrastructure for bus fleets: Evaluation of slow refueling concept for bus depots and estimates of hydrogen supply cost. IFE/E (2020)

[23] Caponi, R., Ferrario, A.M., Del Zotto, L., Bocci, E.: Hydrogen refueling station cost model applied to five real case studies for fuel cell buses. In: E3S Web of Conferences, vol. 312, p. 07010 (2021). EDP Sciences

[24] Swidler, D.S.: Life Cycle Assessment of Hydrogen as a Transportation Fuel in the California Market. ScholarlyCommons (2020). https://core.ac.uk/download/pdf/359025906.pdf

[25] Staffell, I., Scamman, D., Abad, A.V., Balcombe, P., Dodds, P.E., Ekins, P., Shah, N., Ward, K.R.: The role of hydrogen and fuel cells in the global energy system. Energy Environ. Sci. **12**(2), 463–491 (2019)

[26] Parks, G., Boyd, R., Cornish, J., Remick, R.: Hydrogen station compression, storage, and dispensing technical status and costs: Systems integration. Technical report (2014). https://www.nrel.gov/docs/fy14osti/58564.pdf

[27] Caponi, R., Ferrario, A.M., Bocci, E., Valenti, G., Della Pietra, M.: Thermodynamic modeling of hydrogen refueling for heavy-duty fuel cell buses and comparison with aggregated real data. Int. J. Hydrogen Energy **46**(35), 18630–18643 (2021)

[28] Nexant, I., Liquide, A., Argonne National Laboratory, C., Laboratory, P.N.N., LLC, T.: H2A hydrogen delivery infrastructure analysis models and conventional pathway options analysis results. Interim Report to the US Department of Energy (2008). https://www1.eere.energy.gov/hydrogenandfuelcells/pdfs/nexant_h2a.pdf

[29] Stevens, R., Lewis, E., McNaul, S.: Comparison of Commercial, State-of-the-Art, Fossil-Based Hydrogen Production Technologies. NERL (2021). https://www.osti.gov/biblio/1875491

[30] Maack, M.: Generation, of the energy carrier HYDROGEN-In context with electricity buffering generation through fuel cells. Publicerad inom New Energy Externalities Developments for Sustainability (NEEDS) (2008). https://www.yumpu.com/en/document/view/46449402/generation-of-the-energy-carrier-hydrogen-in-context-needs

[31] Wulf, C., Kaltschmitt, M.: Life cycle assessment of hydrogen supply chain with special attention on hydrogen refuelling stations. Int. J. Hydrogen Energy **37**(21), 16711–16721 (2012)

[32] Bonner, B.: Advanced hydrogen fueling station supply: Tube trailers.

Technical report (2018). https://doi.org/10.2172/1469970

[33] Hasan, S., Shabaneh, R.: The economics and resource potential of hydrogen production in saudi arabia. King Abdullah Pet. Stud. Res. Cent. (2021). https://doi.org/10.30573/KS--2021-DP24

[34] OCHA Middle East and North Africa (ROMENA): Saudi Arabia - Subnational Administrative Boundaries. Humanitarian Data Exchange. Accessed 04 Mar 2023 (2021). www.gadm.org

[35] Hoteit, H.: CO2 footprint from industrial facilities in Saudi Arabia. Mendeley Data (2022). https://doi.org/10.17632/mmrtv3nnt7.1

[36] Jufri, F.H., Aryani, D.R., Garniwa, I., Sudiarto, B.: Optimal battery energy storage dispatch strategy for small-scale isolated hybrid renewable energy system with different load profile patterns. Energies **14**(11), 3139 (2021)

[37] Khan, M.I.: Comparative well-to-tank energy use and greenhouse gas assessment of natural gas as a transportation fuel in pakistan. Energy Sustain. Dev. **43**, 38–59 (2018)
